# Mechanisms of action, chemical characteristics, and model systems of obesogens

**DOI:** 10.1186/s42490-020-00040-6

**Published:** 2020-04-30

**Authors:** Mallory D. Griffin, Sean R. Pereira, Megan K. DeBari, Rosalyn D. Abbott

**Affiliations:** Carnegie Mellon University, 5000 Forbes Avenue, Scott Hall, Pittsburgh, PA 15213 USA

**Keywords:** Obesogens, Endocrine disrupting chemicals, In vitro models, In vivo models, Obesogenic mechanisms, Hormone interference, Inflammation, Chemical characteristics, Model systems

## Abstract

There is increasing evidence for the role of environmental endocrine disrupting contaminants, coined obesogens, in exacerbating the rising obesity epidemic. Obesogens can be found in everyday items ranging from pesticides to food packaging. Although research shows that obesogens can have effects on adipocyte size, phenotype, metabolic activity, and hormone levels, much remains unknown about these chemicals. This review will discuss what is currently known about the mechanisms of obesogens, including expression of the PPARs, hormone interference, and inflammation. Strategies for identifying obesogenic chemicals and their mechanisms through chemical characteristics and model systems will also be discussed. Ultimately, research should focus on improving models to discern precise mechanisms of obesogenic action and to test therapeutics targeting these mechanisms.

## Background

The economic impact of obesity worldwide is estimated to be 2.8% of the global gross domestic product [1]. While an imbalance in energy intake and expenditure is largely to blame, other factors contribute to this high economic burden. An analysis of obesity trends (the National Health and Nutrition Examination Study) found that from 1988 to 2006 for the same caloric intake and physical activity, the average BMI was higher [2]. Grun and Blumberg in 2006 [3] hypothesized that this increase in average BMI may be related to obesogens, a subset of endocrine disrupting chemicals (EDCs) that alter metabolism to favor lipid storage, leading to a predisposition to obesity. These chemicals can be found in pesticides, cleaning products, and food and beverage packaging [4]. Regular exposure to these contaminants can have long-term effects on adipose tissue, metabolic activity, hormones, and ultimately weight. Additionally, prenatal exposure may put people at risk for becoming obese later in life. As obesity care is a billion-dollar industry worldwide, identifying and understanding these obesogens is a crucial step in reforming worldwide health.

This review discusses possible mechanisms of obesogenic action including regulation of the PPAR genes, hormone interference, and inflammation. Insights into mechanisms of obesogens will allow for specific therapeutic targeting to minimize effects and aid in predicting potential obesogens from environmental contaminants. This review also discusses advantages and disadvantages of current model systems that are being used (both in vitro and in vivo as well as epidemiological studies) to study obesogens.

## Main text

### Chemical characteristics of obesogens

**Fig. 1:**
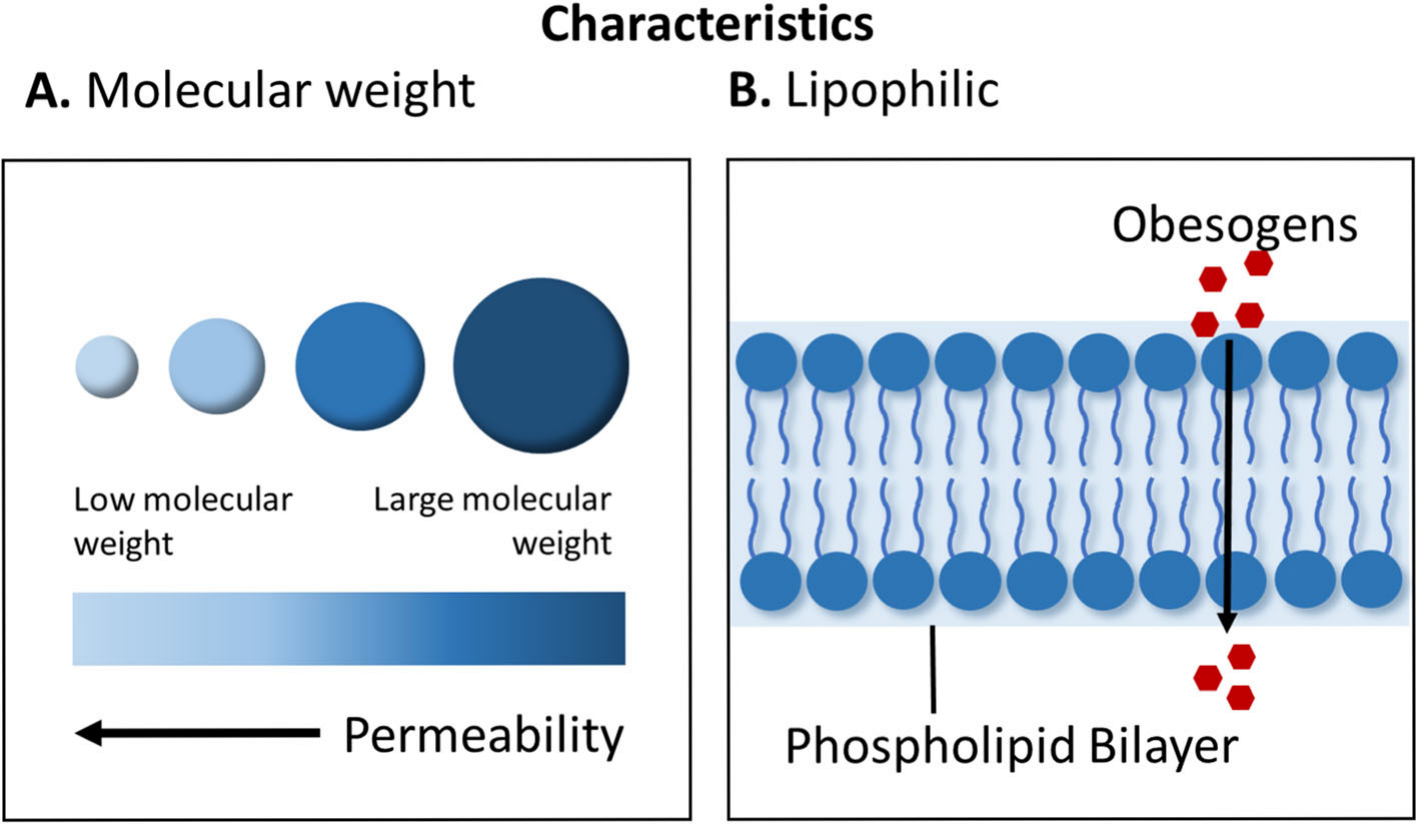
Obesogens have characteristics that make them well-suited to enter cells including small molecular weight (**a**) and lipophilicity (**b**). These properties allow obesogens to easily pass through cell membranes to influence cellular responses and gene expression

Obesogens work through a diverse set of mechanisms [5]. They have been known to mimic or partially mimic natural hormones, having undesired biological effects [6]. They can bind to receptors in the cell membrane, cytosol, or the nucleus affecting cellular responses, peptide hormones, or gene expression directly [6]. Their ability to do this depends on having chemical characteristics that resemble natural hormones including lipophilicity and small molecular weight (Fig. 1). Three key properties that may influence the ability of obesogens to act as xenohormones are the partition constant, half-life, and molecular weight. The partition constant is an equilibrium constant that measures how a compound distributes between two immiscible solvents. The octanol:water partition coefficient (K_ow_) is the ratio of a compound’s partition (divide) between organic matter and water [7]. The equation for K_OW_ is defined as: concentration of chemical in octanol phase / concentration of chemical in aqueous phase [7]. The equation gives a measure of how a chemical will split between tissue and serum at equilibrium. As lipids are organic matter, it is thus an accepted measure of the lipophilicity of the compound. A higher K_OW_ indicates a more lipophilic substance and a propensity to accumulate in adipose tissue [6, 7]. The biological half-life of a chemical is the time it takes for half of the amount of the chemical to be broken down or removed from the body. A longer biological half-life indicates longer persistence in the body. This is particularly relevant to obesogens as a longer biological half-life can mean even a brief exposure can have long-term effects [8]. Molecular weight is a measure of the size of the compound. This is important, since smaller molecules can diffuse into adipocytes more easily. Additionally, even high molecular weight chemicals can be broken down into low molecular weight metabolites in the body that can have obesogenic effects [7]. These three properties tend to have a profound effect on accumulation in the body and affinity for receptors [7, 9]. Lipophilic substances with low molecular weights cross cell membranes easily [6]. Those with long biological half-lives can reside in adipose tissue for months to years. Many well-studied obesogens fit these criteria. A short list of established obesogens and their molecular characteristics are given in Table 1. Lipophilic compounds are also more resistant to degradation, leading to many of them having a biphasic half-life such as 2,2′,4,4′-tetrabromodiphenyl ether (BDE-47) (Table 1) [6]. Substances that are biphasic have an elimination curve that is steep that describes the initial distribution of the drug in the body, followed by shallow curve that describes the final removal of drug, which is dependent on the release of the drug from tissue compartments such as adipose tissue into the blood [20]. Obesogens also have a strong affinity for receptors in the body, specifically nuclear receptors. This could be attributed to the lipophilic nature of the compounds that resemble steroid substances found heavily in adipose tissue [21]. However, more studies need to be done to find other physiochemical properties that control EDCs ability to utilize these receptors.

**Table 1:** Sample list of a subset of well-established obesogens with their partitioning coefficients, half-life, and molecular weight (g/mol). The octanol/water partitioning coefficient is measured using the log K_ow_ value, which indicates the partitioning of a chemical between octanol and water. In these experiments, octanol is used to mimic fat and other hydrophobic components in biological systems. A partitioning coefficient level greater than 1 indicates low solubility in water

Obesogen Source	Obesogens^**a**^	Chemical Characteristics*		
		Partitioning (Log K_ow_) ^b^	Half-life (hours)^c^	Size (g/mol)
Industrial Chemicals	Bisphenol A (BPA)	**3.32**	5.3^d^ [10, 11]	228.291
	Bisphenol A diglycidyl ether (BADGE)	**~ 3.84**	120^e^ [12]	340.419
	Bisphenol S (BPS)	**~ 1.65**	6.93 [13]	250.268
	Firemaster 550 (FM550)	**8.80–11.95**^**f**^	Unknown [14]	549.923
	2,2′,4,4′-Tetrabromodiphenyl ether (BDE-47)	**6.81 ± 0.08** [15]	**664 days**^**g**^	485.795
	3,3′,4,4′-Tetrachlorobiphenyl (PCB-77)	**6.72**	152**–**186 [16]	291.980
	Mono-(2-ethylhexyl) phthalate (MEHP)	**4.92**^**h**^	4.4–6.6^i^	278.348
	bis(2-ethylhexyl) phthalate (DEHP)	**7.60**	**5**^**j**^	390.564
Biocides	Dichlorodiphenyl-trichloroethane (DDT)	**6.91**	**10.6**^**k**^	354.476
	Tributyltin (TBT)	**3.90–4.90** [17]	**23–30 days** [18]	290.058
	Triphenyltin (TPT)	**4.19**^**l**^	**3 days**	385.478
Pharmaceuticals	Diethylstilbestrol (DES)	**5.07**	2**–**3 days [19]	268.350
	Estradiol (Estrogen steroid Hormone)	**4.01**	3 days	272.388
Pollutant	Dioxin	**6.80**	**5–8 years**	321.970
Smoking	Nicotine	**1.17**	**1–4**^**m**^	162.236

Sources

^*^Values of partition coefficients and half-lives might differ from those in this table because of variations in the study such as the location the study was conducted, type of tissue, biphasic pattern, initial dosage, temperature, salinity and pH.

^a^The obesogens discussed are well-established obesogens that have been used in various studies.

^b^All values of log K_ow_ were reported at 25°C and at a pH of 7 unless stated otherwise.

^c^All estimated half-life values reported were conducted on studies in human based models unless stated otherwise.

^d^BPA data are not consistent with the current consensus that BPA exposures are both rapidly cleared and almost entirely related to food intake. Instead, it appears plausible that there is substantial nonfood exposure, accumulation in body compartments with long elimination times, or both [3].

^e^Bisphenol A diglycidyl ether based on Hydrolysis in Water.

^f^2-ethyl-1-hexyl-2,3,4,5-tetrabromobenzoate (TBB) and bis (2-ethylhexyl) tetrabromophthalate (TBPH) are the two major additive Brominated flame retardants (BFRs) in Firemaster 550. [16].

^g^Tetrabromodiphenyl ether (BDE-47) has two phases of elimination. The first phase of elimination is where the majority of the BDE-47 is eliminated from the body (67%), and the remaining BDE-47 is eliminated during the terminal phase. Additionally, elimination, both whole-body and from individual tissues, is biphasic due to varying initial and terminal phase lengths in different tissues. Since BDE-47 is highly lipophilic its terminal phase was primarily dictated by adipose tissue and skin [21].

^h^MEHP was estimated from n-octanol: water coefficient (K_ow_) by the algorithm from Poulin and Krishnan (1993). A log K_ow_ of 4.92 was estimated based on the chemical structure for nonionized MEHP.

^i^Single administration of MEHP in a rat (0.4 g/kg) resulted in plasma concentrations of 84.1 +/− 14.9 micrograms/ml 3 h after dosing; the half-life of MEHP was 5.5 +/− 1.1 h. [22].

^j^After an absorption and distribution phase of 4 to 8 h, half-life times of excretion in the first elimination phase were approximately 2 h; Half-life times in the second phase—beginning 14 to 18 h post dose—were 5 h for MEHP [23].

^k^Female rats were dosed orally with (14) C-ring-labeled p,p’-DDT during pregnancy or lactation. Average half-life was 10.6 h in tissues and in the fetus.

^l^Triphentylin Chloride was used to find the log K_ow_.

^m^Nicotine’s half-life in the initial phase is reportedly about 2–3 min and the half-life in the terminal phase averages about 2 h.

Note: All partitioning coefficient values > 1 indicate lipophilic properties (bold). Half-life with strong indication of biphasic pattern (bold). Non-asterisk and cited chemical characteristics were obtained from the U.S. National Library of Medicine Open Chemistry Database and the International Programme on Chemical Safety.

### Mechanisms of action of obesogens

**Fig. 2:**
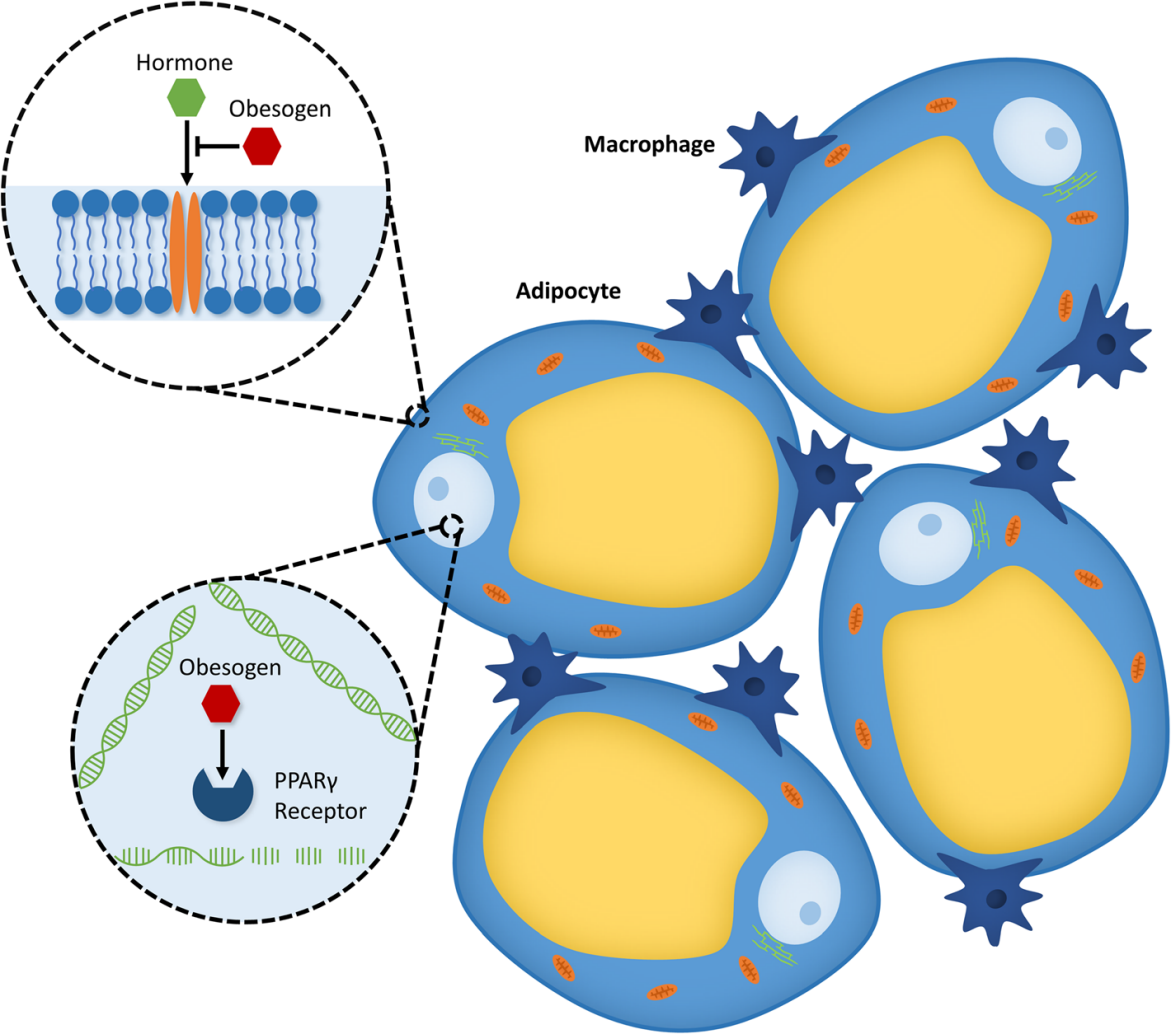
Obesogens can interfere with endocrine function by direct hormone interference or activation of peroxisome proliferator-activated receptor gamma (PPARγ) in adipocytes. Obesogens can also alter appetite and satiety through neuroendocrine mechanisms

Definitive mechanisms for obesogens are still in the early stages of investigation. Current research points to a major role of peroxisome proliferator-activated receptor gamma (PPARγ), hormone interference, and inflammation in obesogenic outcomes (Fig. 2). While the role of these three mechanisms in obesogenic effects will be discussed in the following section, it should be noted that many potential mechanisms for obesogens exist, not all of which will be discussed here. It should also be noted that there are likely distinct pathways for developmental (in utero) and non-developmental exposures of obesogens, as well as persistent versus non-persistent exposures, with more research required to clearly define these differences. For more comprehensive reviews on what is currently known about mechanisms of obesogens see [22–24].

#### Activation of peroxisome proliferator-activated receptor gamma (PPARγ)

Peroxisome proliferator activated receptors (PPARs) are a group of non-steroid nuclear hormone receptors [25, 26]. There are three known isoforms of PPAR: (1) PPARα; (2) PPARβ/δ; and (3) PPARγ. Each isoform is encoded by a separate gene [26, 27]. PPARs bind with the nuclear receptor 9-cis retinoic acid receptor (RXR) to form their own heterodimers. These heterodimers modulate expression of target genes [28–31]. The heterodimer binds to specific response sites called peroxisome proliferator response elements (PPRE) in the promoter region of target genes. Subsequent binding of a ligand to the receptor then alters the conformation of PPAR to induce recruitment of co-transcription factors. This results in an increase in mRNA expression of the target gene [28, 29, 31, 32]. PPARs target genes related to lipid storage, transport, and metabolism including fibroblast growth factor 1 (FGF1) (PPARγ) [33], G-protein-coupled receptor 81 (*GPR81*) (PPARγ) [34], adiponectin (PPARα), [35], and CPT-1 (PPARα) [36, 37] and so are common targets in the study of obesogenic mechanisms [28, 29, 31].

PPARγ is the most widely studied transcription factor in terms of adipose tissue development and is required for adipogenesis [38–40]. Thiazolidinedione drugs used to treat type 2 diabetes target PPARγ to increase insulin sensitivity with the side effect of inducing adipogenesis [41]. Many obesogens have already been shown to upregulate this gene. Tributyltin (TBT), one of the most widely studied obesogens, activates the PPARγ/RXR heterodimer in vitro [42, 43], in utero [44], and in vivo [43]. It is unclear if the effects are due to the activation of the PPARγ domain itself, the RXR domain, or both. It is likely that TBT activates the PPARγ/RXR complex through binding of the RXR domain since transfected Cos7 cells were activated by TBT in the presence of a PPARγ antagonist [42]. Additionally, commitment of mesenchymal stem cells to the adipogenic lineage has been shown to be dependent on RXR activation and not PPARγ activation [45]. However, further analysis needs to be done to affirm this conclusion. Other obesogens that have also been shown to act at least partially through PPARγ/RXR activation include bisphenol A (BPA, plastic monomer) [46–48], triflumizole (fungicide) [49], phthalate monoesters (plasticizers) [50], Firemaster 550 (flame retardant) [51], and dioctyl sodium sulfosuccinate (DOSS) (component of oil dispersant COREXIT) [52]. It is likely that different obesogens have different mechanisms for activating the PPARγ/RXR heterodimer and further research will be needed to determine specific molecular mechanisms. Understanding the specific effects of these obesogens on the PPARγ/RXR heterodimer may provide valuable insight for reversing obesogenic effects.

PPARα is another isoform of PPAR. It is found primarily in the liver, skeletal muscle, heart, and brown adipose tissue and plays a major role in fatty acid metabolism in the liver [53–55]. Natural ligands include oxidized phospholipids, lipoprotein lipolytic proteins, and fatty acids, among other natural ligands [54]. Although it is found primarily in liver and skeletal muscle, there is increasing evidence for its role in adipose tissue and as a target for obesogens. PPARα is known to improve insulin sensitivity and aid in body weight control in rodents [53, 54]. Agonists have also been shown to reduce insulin resistance and decrease body weight in male mice [53]. PPARα-deficient mice have been shown to have upregulated mRNA expression of adiponectin, but this is thought to be the result of increased adipose tissue mass or an attempt to counterbalance a concomitant increase in leptin expression [56]. PPARα is not as widely studied as PPARγ in terms of obesogens but current literature suggests there is an effect. Monosodium glutamate (MSG) and aspartame decreased gene expression of PPARα in mice [57]. TBT was shown to activate PPARα in transfected HeLa cells [58] and mice exposed to TBT in utero showed increased mRNA expression of PPARα [59]. Since PPARα is known to improve insulin sensitivity, the increased expression could be one of the mechanisms for obesogenic effects. However, the obesogen bis (2-ethylhexyl) phthalate (DEHP) increased mRNA expression of PPARα in liver tissue while decreasing expression in visceral fat in mice [60]. The mechanisms of obesogens are likely more complex than what is currently understood and further research will be required to draw conclusions.

#### Hormone interference

Exogenous chemicals that are capable of mimicking or interfering with hormonal action can have profound effects on the overall function of metabolic processes. Hormones such as androgens and estrogens are tightly regulated and play an important role in the function of adipose tissue. Increasing androgen levels are associated with lower BMIs in men [61]. Several phthalates are suspected antiandrogens [62] and have shown obesogenic effects in humans. BPA acts as a xenoestrogen. When mice are exposed perinatally to BPA, the offspring are significantly heavier [48]. Dichlorodiphenyldichloroethylene (DDE, a metabolite of the common pesticide dichlorodiphenyl-trichloroethane, DDT) has also been shown to exhibit estrogenic responses [63]. It leads to rapid weight gain in infants after prenatal exposure. BPA, phthalates and polybrominated diphenyl ethers (PBDEs) also have been shown to reduce circulating thyroid levels [64], a key regulator of basal metabolism. Decreased thyroid hormone levels result in an increased BMI [65]. Leptin and adiponectin are also influenced by obesogens. Leptin, discovered by Zhang et al., is responsible for satiety and increases glucose uptake by skeletal muscle and brown adipose tissue [66, 67]. Mutations in leptin result in obesity and hyperinsulinemia. However, hyperleptinemia, as is common in obesity, can lead to leptin resistance [66, 68]. Adiponectin, first discovered by Scherer et al., is known to increase insulin sensitivity [69, 70]. Multiple obesogens have been shown to have an effect on these hormones. TBT increases plasma leptin levels in mice, causing an overexpression of the leptin gene, and decreased serum adiponectin levels [71, 72]. DEHP decreases both adiponectin and leptin mRNA levels in mice [60]. DOSS increases plasma leptin levels in male mice exposed in utero [73]. Genistein, an isoflavone found in soy, induced adipose deposition in male mice, increased insulin resistance, and upregulated mRNA expression of leptin [74]. DEHP has also been shown to increase serum leptin levels [75]. Benzyl butyl phthalate (BBP, plasticizer) was shown to increase adiponectin protein expression in differentiated 3T3-L1 cells [76]. Additionally, glucocorticoid receptor signaling is crucial for adipocyte differentiation [77]. Sargis et al. [78] demonstrated increased adipogenic differentiation via glucocorticoid receptor activation with BPA, dicyclohexyl phthalate (DCHP), endrin, and tolylfluanid (TF). Hormones are a common target of obesogens but the exact effect of each obesogen and the mechanisms of hormone influence are yet to be determined. Moreover, there are likely other hormonal targets that are still unidentified.

#### Inflammation

Obesity is associated with chronic inflammation. While inflammation is associated with adipose tissue expansion, it may also be the result of epigenetic changes due to environmental and lifestyle factors [79]. DOSS has been shown to increase body mass, visceral fat mass, upregulate inflammatory gene expression (Cox2, Nox4), and increase plasma levels of IL-6 in male mice exposed in utero [73]. Likewise, TBT exposure in rats upregulated PPARγ, increased ovarian fat mass, and increased reproductive tract inflammation in rats [80]. A similar study in female rats showed increased body weight and uterine inflammation after TBT exposure [81]. Male mice exposed to BPA showed increased gene expression of IL-6, TNF-α, and IL-1β in white adipose tissue and increased fat mass on a chow-diet [82]. Differentiated 3T3-L1 preadipocytes also show increased expression of IL-6, TNF-α, MCP-1, and CXCL1 after exposure to either TBT, BPA, or mono-ethylhexyl phthalate (MEHP, metabolite of DEHP) [83]. Moreover, a study on male mice showed an Il-17 antibody was able to reduce inflammation and counter the obesogenic effects of BPA, suggesting inflammation plays a major role in the obesogenic effects of BPA [84]. Multiple obesogens have also been shown to increase the presence of immune cells in adipose tissue. Female sheep exposed to BPA show increased mRNA expression of CD68, a marker of macrophage infiltration [85]. Additionally, mice exposed to BPA perinatally showed increased presence of macrophages in gonadal white adipose tissue [86]. BPA has also been shown to increase macrophage self-renewal [87]. BPA is one of the most widely studied obesogens but it is likely that an influx of inflammatory cells plays a role in other obesogens as well. There is also evidence for a correlation between the PPAR genes and inflammation. While they are upregulated during inflammation they also act as negative feedback loops by being antagonists to transcription factors for proinflammatory genes [88–92]. Antidiabetic drugs, such as thiazolidinediones antagonize tumor necrosis factor-α (TNF-α) [93] and act as agonists for PPARγ [94]. This area is in early stages of research but suggests a role for inflammatory cells and gene expression in obesogenic modes of action.

### Model systems

Currently, model systems are used to test mechanisms of obesogenic action including in vitro and in vivo systems as well as epidemiological studies. Each type poses unique benefits and drawbacks to establishing mechanisms. Common systems for each type are discussed below along with advantages and disadvantages.

#### *In vitro* models

In vitro models pose several benefits over other model systems. They can utilize human cell types to be more physiologically relevant. They are also generally simpler, faster, can be done in parallel (for medium to high throughput analyses), and are more cost-effective, making them a good screening mechanism for obesogens prior to in vivo studies. Currently, there are several in vitro models to screen potential obesogens that examine characteristics such as adipocyte maturation and lipid accumulation (Table 2). The vast majority of these models utilize mouse 3T3-L1 preadipocytes. These cultures have been integral in elucidating certain molecular mechanisms of adipogenesis. However, it is still unclear if the 3T3-L1 cell line is adequate for evaluating adipogenic responses, since they are fully committed to the adipocyte lineage [120, 121]. Additionally, the murine-derived 3T3-L1 cell line maintains species specificity, which may hinder application of results for human-based risk assessments. Use of human primary cell lines mitigates this risk but further limitations exist. Patient demographics and medical histories are unknown to researchers and contribute large variability in outcomes [122]. Sex specific differences are often not accounted for and gender is known to dictate body fat storage [123] and responses to obesogens [48]. Future work needs to work on validating these models using primary cells or tissues from a wide range of known patient demographics. There are also depot-specific effects of obesogens on adipose tissue. Cells derived from visceral versus subcutaneous or brown versus white adipose tissue may have varying responses to obesogens. As visceral adipose tissue is most closely linked to metabolic disease, understanding differential responses by adipose tissue depots is crucial for defining obesogenic effects.

**Table 2:** 2D and 3D in vitro models for studying obesogens. Note: -- under *Matrix* indicates a 2D cell culture study

Matrix	Cell Type	Obesogen	Source
–	3T3-L1 (murine preadipocyes)	Tributyltin (TBT)	[42, 43, 94–99]
		Bisphenol A (BPA)	[97–99]
		Bisphenol S (BPS)	[102]
		Bisphenol A diglycidyl ether (BADGE)	[100]
		Triphenyltin	[95]
		Dioctyl sodium sulfosuccinate (DOSS)	[52]
		Geneistein & naringenin	[103]
		Phthalate monoesters	[32]
		4-nonylphenol (NP)	[101]
		Mono-ethylhexyl phthalate (MEHP)	[50]
		Flavanone	[104]
		Bixin, norbixin	[105]
		Emodin	[106]
	C2C12 (murine)	Mono-ethylhexyl phthalate (MEHP)	[50]
	HELA (human)	Mono-ethylhexyl phthalate (MEHP)	[50]
	Human embryonic kidney cells (HEK293C)	Dioctyl sodium sulfosuccinate (DOSS)	[52]
	HepG2 (human liver carcinoma cells)	Bisphenol A (BPA), Bisphenol S (BPS)	[102]
	Human adipose-derived stem cells (hASCs)	Bisphenol A (BPA), Bisphenol A diglycidyl ether (BADGE)	[102]
		Tributyltin (TBT)	[44]
	Murine adipose derived stem cells (mASCs)	Bisphenol A (BPA), Bisphenol A diglycidyl ether (BADGE)	[102]
		Tributyltin (TBT)	[44]
	Fao (murine hepatoma cells)	Phthalate monoesters	[32]
	COS (monkey kidney-derived cells)	Bisphenol A (BPA), Bisphenol A diglycidyl ether (BADGE)	[102]
		MBzP, MBuP	[50]
		Mono-ethylhexyl phthalate (MEHP)	[50, 107]
	THP-1 macrophages (human)	Psi-baptigenin, hesperidin	[108]
	TARM-Luc (human, transfected T47-D epithelial cells)	Monosodium glutamate (MSG)	[109]
	KS483 (murine calvaria)	Soy phytoestrogen genistein	[110]
	C57BL/6 (murine–derived bone marrow stromal cells)	Firemaster 550	[51]
collagen embedded silk scaffolds	Human embryonic-derived stem cells (hESCs)	Tributyltin (TBT), Bisphenol A (BPA), Bisphenol S (BPS)	[111]
silk scaffolds	Human adipose-derived stem cells (hASCs), Human umbilical vein endothelial cells (HUVECs)	–	[112]
silk fibroin matrices	Human adipose-derived stem cells (hASCs)	–	[113]
collagen type 1	OP9 (murine mesenchymal stromal pluripotent cells), HaCaT (human keratinocytes)	Super Hatomugi (SPH)	[114]
bacterial nanocellulose	Murine mesenchymal stem cells (mMSCs)	–	[115]
fibrous polyethylene teraphthalate scaffolds	3T3-L1 (murine preadipocyes)	–	[116]
low-shear rotary bioreactor	Murine adipose-derived stem cells (mASCs)	–	[117]
polyglycolic acid fiber meshes	3T3-L1 (murine preadipocyes)	–	[118]
adipospheres created via magnetic nanoparticle levitation system	3T3-L1 (murine preadipocyes)	–	[119]

To better understand the impact of obesogenic chemicals in more physiologically relevant environments, scientists have been examining 3D human tissue systems to model the effects of obesogens in vitro (Table 2). 3D adipose tissue systems recapitulate the in vivo adipose tissue microenvironment, can be extended for long term culture [122, 124] (months, to study chronic effects of obesogens) and can incorporate multiple cell types. They can be used to study the sequestration of obesogens in adipose tissue as well as cell migration. Obesogens are primarily lipophilic and thus prone to retention by adipose tissue [125]. 3D models can incorporate mature adipocytes which are non-adherent and cannot be cultured using standard 2D culture techniques. Similarly, they allow for long-term in vitro study of ASC differentiation which also become non-adherent over time [126]. The use of 3D models allows for more sophisticated co-culture systems. As multiple organs play a role in obesogenic actions including adipose tissue, pancreas, liver, thyroid, etc., systems integrating multiple cell types may provide more physiologically accurate data. They can also study paracrine signaling. However, 3D models increase cost and complexity over 2D systems, since they use natural or artificial extracellular matrices (ECM). This brings the added variables of cell binding domains, mechanical properties, pore size, etc. Perfusion cultures also pose issues related to flow rates, media, and fluid/cell ratios. Finally, most in vitro studies are currently 2D which limits the ability to compare results from 3D cultures to already-established models. Overall, both 2D and 3D in vitro models provide precise control of cellular interactions and boundary conditions, permitting quantitative analyses of mechanisms. They are ideally suited for high-throughput screening as they can test dose responses and mixture effects in parallel. While in vitro models have limitations that must be resolved, they provide strong screening potential for obesogens.

#### *In vivo* models

Animal models have the distinct and obvious disadvantage of not accurately replicating human physiology. However, animal models are an important and widely used tool for the study of obesogens because they are suited for studying whole body kinetics and systemic effects not possible in vitro. Metabolism and weight is regulated by complex interconnected pathways involving multiple organs including adipose tissue, liver, pancreas, muscle, brain, etc. [127]. Although in vitro cell culture techniques can use human cell lines, recapitulating the inter-dependency of these systems remains difficult. Long-term in vitro culture remains a challenge and multi-organ models pose unique problems such as scaling ratios, common mediums, and organ-specific ECMs. Thus, although more sophisticated in vitro models are being heavily researched, animal models still play an important role in identifying obesogens and understanding obesogenic mechanisms because they allow for the study of organ cross-talk and systemic effects. This is particularly relevant in understanding the role of chronic inflammation and hormone interference.

Rodents are the most commonly used animal model for studying obesogens. Multiple obesogens have been identified using murine models including: TBT [43], BPA [82], triphenyltin [43, 95], DEHP [128], DES [129], MEHP [130], polycyclic aromatic hydrocarbons [131, 132], DDT [133], and nicotine [134]. Mice are biologically and anatomically similar to humans and contract many of the same diseases [135]. This is particularly useful for diseases with an inflammatory component, such as obesity [136], as animal models can mimic complex inflammatory responses. Mice can also be genetically manipulated, inbred to yield genetically identical strains, can be grown under controlled conditions (i.e high-fat/western diet), and have an accelerated lifespan (minimizing the time required to do studies). Other common in vivo systems used to evaluate obesogens include: rats, [137–139] zebrafish, [140–142] and the *Xenopus laevi* [143]*.* Use of in vivo models to study endocrine disruption has provided many insights into potential obesogens and different modes of action. However, it is important to keep in mind the drawbacks of using animal models. As discussed, they do not necessarily recapitulate human physiology [144]. Moreover, the dose-response may not translate directly to humans. The window of exposure may also be unnatural. Mice exposed to a specified level of one particular chemical over the course of weeks may not represent chronic fluctuating exposure to multiple chemicals over the course of years in humans. Animal models play an important role in identifying obesogens and discerning mechanisms of action but should be combined with information from in vitro studies and epidemiological studies to draw the most accurate conclusions.

#### Epidemiological studies

Epidemiological studies are extremely important for correlating disease outcomes to concentrations of obesogens in humans. However, human studies linking EDCs and obesity are limited, inconsistent, and lack data to support the growing animal literature (for review see Hatch et al.*,* 2010 [145]). Current studies are often cross-sectional and exploratory.

Since a significant amount of evidence suggests that prenatal exposures predispose patients towards obesity, measurements of obesogens during pregnancy is a large focus for epidemiological studies. A study revealed that increasing maternal urinary phthalate concentrations during pregnancy doubled the likelihood of the offspring being overweight or obese [146]. Likewise, cohort studies on the effects of prenatal exposures to BPA showed an association with an increased waist circumference, BMI, and risk of being obese [147]. Future work is necessary to compare results from developmental exposures to exposures later in life. Perspective long term studies are also necessary to track patients over time.

Some epidemiological studies examine single-spot urine or 24-h urine samples in order to assess exposure over a day [148]. This method allows investigators to directly measure individual chemical concentrations in a variety of biospecimens [149]. However, it is not possible to determine whether exposures were acute or long term. Although chemical exposure biomarkers have been advantageous in studying a variety of individual biospecimens, short half-lives in certain obesogens (such as BPA that persists as BPA-G) and reverse causality due to pharmacokinetic variables have limited their usefulness [150]. Additionally, given the lipophilic nature of obesogens, it is unlikely that urine samples are an accurate reading of exposure. Ideally, adipose tissue would be sampled directly.

Another important consideration brought up by Sharpe and Drake [151], is the influence of confounding factors in epidemiological studies. They warn that obesogenic exposures may not have a causal relationship with obesity, since a Western style diet increases exposure to these compounds, and thus the observed increase in levels would be reflective of greater food consumption. Another confounding factor is that many epidemiological studies are designed to assess the impact of a single chemical without accounting for the effects of mixtures [148]. Statistical models must be developed in order to elucidate the health outcomes associated with specific chemicals in the mixtures. These studies should account for the fact that some of these chemicals may operate by the same mechanism, while interfering with other mechanisms.

## Conclusion

Abundant evidence supports the role of exogenous chemicals in rising obesity rates through regulation of gene expression (such as the PPARs), hormone changes, and inflammation. A greater understanding of obesogenic mechanisms will lead to better prophylactic and therapeutic strategies and identify other potential obesogens. In vitro models are useful screening tools for identifying and testing mechanisms of obesogens. Specifically, they can help discern changes to gene expression or molecular pathways that induce changes to adipocyte phenotype. Improvements to these models will also improve in vitro to in vivo extrapolation to humans. Still, animal models remain a useful and generally physiologically accurate tool for testing inter-organ obesogenic mechanisms including hormone interference and inflammation. To validate in vitro and in vivo animal models, more comparisons should be made to epidemiological studies. Epidemiological studies provide unparalleled insight into human obesogen exposures and effects. They can be used to identify exposure levels of potential obesogens and to analyze correlative effects between exposure level and BMI, adiposity, leptin/adiponectin levels, etc. This can help determine if there are safe levels of exposure to specific levels or whether drastic actions should be taken to remove a compound entirely. Integrating the information obtained from all three of these model systems will lead to better-informed choices of compounds that can be used in food processing, packaging, etc. to replace obesogens. Ultimately, this will decrease the economic burden of obesity.

## Data Availability

Not applicable.

## Abbreviations

PPAR: Peroxisome proliferator-activated receptor
PPARγ: Peroxisome proliferator-activated receptor gamma
PPARβ/δ: Peroxisome proliferator-activated receptor beta/delta
PPAR⍺: Peroxisome proliferator-activated receptor alpha
EDC: Endocrine disrupting chemical
K_OW_: Partition coefficient
PPRE: Peroxisome proliferator response elements
RXR: Retinoic acid receptor
FGF1: Fibroblast growth factor 1
*GPR81*: G-Protein-coupled receptor 81
BDE-47: 2,2′,4,4′-tetrabromodiphenyl ether
BPA: Bisphenol A
TBT: Tributyltin
DOSS: Dioctyl sodium sulfosuccinate
MSG: Monosodium glutamate
DEHP: Di-2-ethylhexyl phthalate
BMI: Body mass index
DDE: Dichlorodiphenyldichloroethylene
DDT: Dichlorodiphenyl-trichloroethane
PBDEs: Polybrominated diphenyl ethers
BBP: Benzyl butyl phthalate
IL-6: Interleukin-6
TNF-⍺: Tumor necrosis factor-alpha
IL-1β: Interleukin-1 beta
MCP-1: Monocyte chemoattractant protein-1 CXCL1: C-X-C Motif Chemokine Ligand 1
MEHP: Mono-(2-ethylhexyl) phthalate IL-17: Interleukin-17
BPA-G: Bisphenol A β-D-glucuronide
FM550: Firemaster 550
DES: Diethylstilbestrol
DCHP: Dicyclohexyl phthalate
TF: Tolylfluanid
